# Insights into potential causes of vascular hyperpermeability in dengue

**DOI:** 10.1371/journal.ppat.1010065

**Published:** 2021-12-09

**Authors:** Andrew Teo, Caroline Lin Lin Chua, Po Ying Chia, Tsin Wen Yeo

**Affiliations:** 1 Lee Kong Chian School of Medicine, Nanyang Technological University, Singapore, Singapore; 2 Department of Medicine, The Doherty Institute, University of Melbourne, Melbourne, Australia; 3 School of Biosciences, Faculty of Health and Medicine Sciences, Taylor’s University, Subang Jaya, Malaysia; 4 National Centre for Infectious Diseases, Singapore, Singapore; 5 Department of Infectious Diseases, Tan Tock Seng Hospital, Singapore, Singapore; University of Iowa, UNITED STATES

Dengue is a mosquito-borne disease caused by dengue virus (DENV), where four serotypes can infect humans. Most DENV infections are self-resolving, but in some individuals, severe dengue characterised by a sudden increase in haematocrit, rapid decrease in platelet counts, and vascular leakage is a complication [1,2]. In severe dengue, a major pathogenic mechanism is a transient increase in vascular permeability resulting in severe plasma leakage (herein referred to vascular hyperpermeability) leading to hypotension, circulatory collapse, and organ dysfunction [1]. The precise mechanism in DENV-associated vascular hyperpermeability is unclear, and several hypotheses including antibody-dependent enhancement (ADE) and “cytokine storm” have been proposed. In ADE, suboptimal DENV neutralising antibodies against a heterologous serotype (in secondary infection) promotes DENV uptake into immunological cells, increasing infection and viral replication that can exacerbate the immune response [3]. Similarly, infected monocytes release excessive amounts of proinflammatory cytokines and, if dysregulated, can lead to “cytokine storm” [4]. In this article, we present current understandings on the potential causes of dengue-associated vascular hyperpermeability, which is a consequence of complex interactions between the virus and the host endothelium immune response.

## 1. Compromised endothelial glycocalyx integrity contributes to vascular hyperpermeability

**Fig 1:**
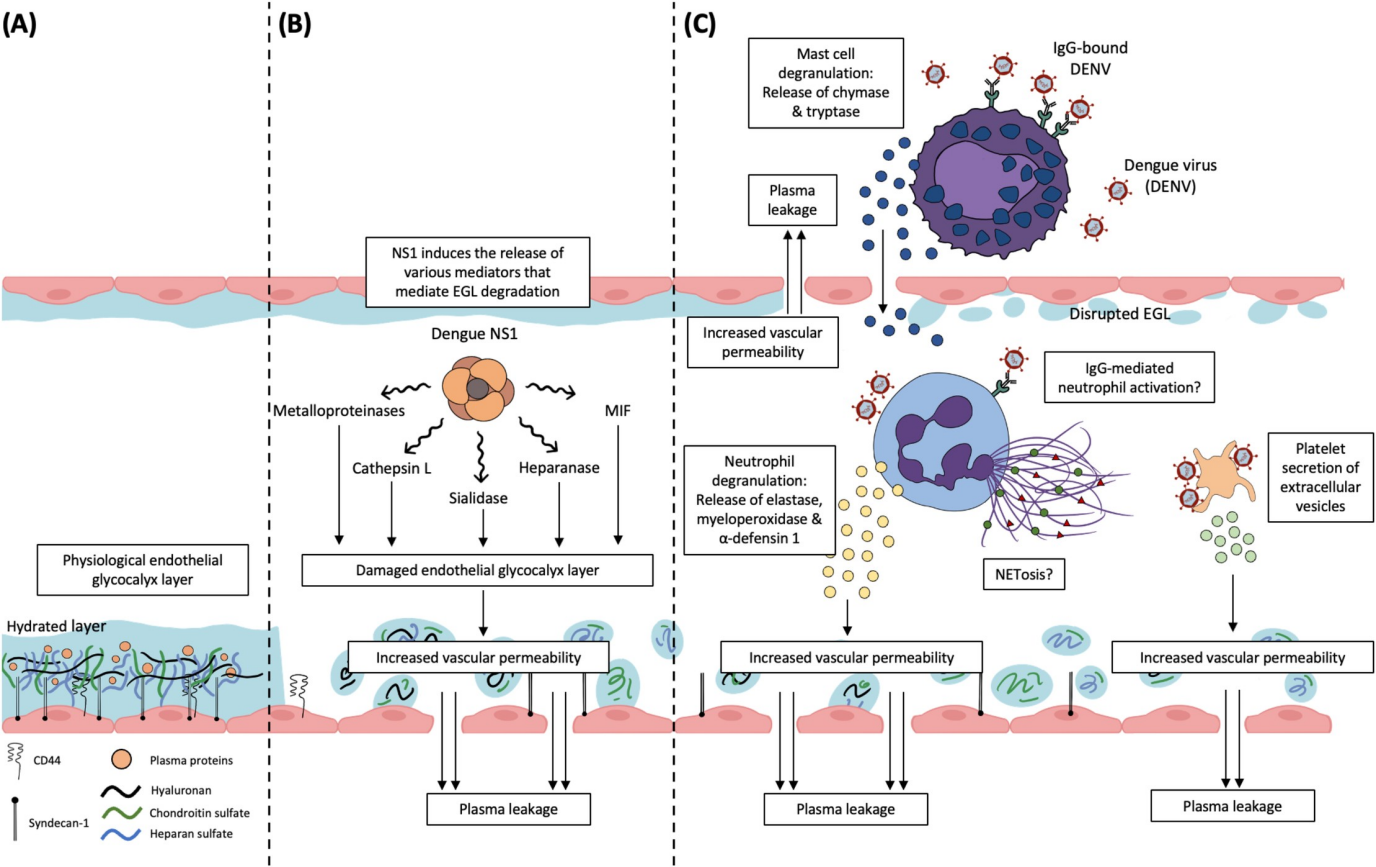
Schematic representation of the EGL and potential EGL disruption during DENV infection. (A) In a healthy blood vessel, the EGL comprising of complex proteoglycans such as syndecans, HS, and CS can be found on the endothelium. This carbohydrate-rich layer plays an important role in maintaining vascular integrity. (B) In DENV infection, NS-1 triggers the expression and activation of various enzymes that can cleave components within the EGL, leading to loss of EGL integrity and subsequent increased vascular permeability. (C) Other potential mechanisms that link immune cells to increased vascular permeability in DENV infection have also been proposed. Activated neutrophils in dengue patients can release granular proteins such as elastase, myeloperoxidase, and α-defensin 1, which are associated with vascular leakage. NETosis by neutrophils has also been reported in DENV infection; however, the mechanism linking this process to vascular permeability is unknown. Similarly, the activation of MCs leads to degranulation and release of potent mediators such as chymase and tryptase. These proteases were found at increased levels in patients with increased severity of vascular leakage, implying their role in vascular pathology. DENV-activated platelets and EVs released are likely to interact with neutrophils, monocytes, and macrophages, resulting in intensified inflammation that contributes to vascular leakage. In patients with DSS, these mechanisms may be acting in concert to contribute to vascular hyperpermeability. CS, chondroitin sulphate; DENV, dengue virus; DSS, dengue shock syndrome; EGL, endothelial glycocalyx layer; EV, extracellular vesicle; HS, heparan sulphate; IgG, immunoglobulin G; MC, mast cell; MIF, macrophage inhibitory factor; NS-1, nonstructural protein-1.

The endothelial glycocalyx layer (EGL) is a glycosaminoglycan-rich barrier, 0.5 to 5.0 μm thick, which coats the luminal surface of the endothelium, forming a mesh that acts as a macromolecular sieve across cell–cell junctions. This surface matrix is composed of various proteoglycans tethered to the underlying endothelial cell including transmembrane syndecans and glycosaminoglycans such as heparan sulphate (HS), chondroitin sulphate (CS), and hyaluronic acid (HA) (Fig 1A). Together with bound plasma proteins such as albumin, fibrinogen, and orosomucoid, the EGL forms a physiological barrier between the intravascular compartment and the interstitium, and damage and degradation may increase vascular permeability [5,6].

Recent studies suggest that EGL is compromised during dengue. EGL disruption and hyperpermeability was reported in dengue mouse model [7,8], and, using videomicroscopy, the thinning of the sublingual EGL was observed in dengue patients with vascular hyperpermeability [9]. Furthermore, increased blood levels of HA, syndecan-1 and CS in dengue patients were proportional to disease severity, further suggesting that EGL is damaged in clinical disease [5,6,10]. Importantly, EGL degradation has also been linked to coagulation disorders, increased leukocyte adhesion to the endothelium, and fluid extravasation, which are associated with increased plasma leakage [9–11]. The precise mechanisms that trigger the shedding of EGL components in DENV infection remains elusive.

## 2. Dengue nonstructural protein 1: A mediator of vascular hyperpermeability

DENV nonstructural protein-1 (NS1) is a highly conserved 48-kDa membrane-associated glycoprotein. Interestingly, NS1 is the only nonstructural viral protein secreted by infected host cells [12], and circulating levels of NS1 correlated positively with vascular permeability in vitro and in vivo [5,8,13,14]. However, other clinical studies have not shown a correlation between NS-1-induced vascular permeability. These highlight the complexity of DENV-mediated vascular pathologies and differences in in vitro NS-1 quantification, and the timing of sampling likely contributed to the discrepancies [14–17]. Recent in vitro studies have demonstrated that NS1 protein (expressed by all four DENV serotypes) can directly promote the expression and activation of sialidase, cathepsin L, and heparanase, with the latter enzyme subsequently inducing syndecan-1 shedding to cause vascular hyperpermeability [7,8]. This mechanism of EGL breakdown occurred independently of inflammatory mediators [7].

NS1 also interacts with Toll-like receptor (TLR) 4 and induces the production of proinflammatory and vasoactive mediators including cytokines and vascular growth factors, many of which are likely to cause damage to EGL and inducing vascular pathology [18–20]. Two noteworthy molecules induced by NS1 are matrix metalloproteinases (MMPs) produced by leukocytes and macrophage inhibitory factor (MIF) secreted by endothelial cells [13,21]. EGL components such as syndecan-1, syndecan-2, and syndecan-4 can be cleaved by MMP-9, and this process may be further enhanced through MIF. In DENV infection, increased levels of MMP-2, MMP-9, and MIF have been associated with increased risk of dengue severity, and both MMP-9 and MIF were reported to induce vascular hyperpermeability [13,21,22] (Fig 1B). Additionally, NS-1-induced MMP-9 was demonstrated to disrupt junctional adhesion molecules in vitro, which may trigger vascular hyperpermeability [13]. In a murine dengue model, inhibition of MIF abolished NS-1-induced MMP-9 secretion, and this correlated with reduced syndecan-1 shedding, suggesting that MIF is an important factor that mediates vascular pathology in DENV infection [21]. Overall, these studies suggest that specific targeting of DENV-NS1 protein may have therapeutic potential to prevent EGL breakdown.

## 3. Activated neutrophils contribute to vascular hyperpermeability

Neutrophils are the most abundant leukocytes in the peripheral circulation. In vitro exposure of neutrophils to DENV resulted in the secretion of leukotriene B4, a potent neutrophil chemoattractant, suggesting neutrophil recruitment during DENV infection [23]. In an immune profiling study, increased genetic signature of neutrophil-associated transcripts in the early phase of acute dengue was associated with progression towards dengue shock syndrome (DSS), a consequence of vascular hyperpermeability [24]. Similarly, overexpression of genes encoding for several neutrophil granular proteins were observed in DSS cases compared to mild dengue cases, highlighting neutrophils involvement in severe dengue [25]. Neutrophils are densely packed with potent antimicrobial secretory granules, and stimulation of these immune cells with DENV can cause degranulation and neutrophil extracellular traps (NETs) formation; these were associated with increased vascular permeability (Fig 1C). In dengue patients, a significant increase in the peripheral circulation of canonical proteins that are associated with neutrophil degranulation, and granular proteins such as neutrophil elastase and α-defensin 1 were observed in DSS cases compared to either healthy controls or uncomplicated dengue cases [26–28]. Furthermore, NETs formation (quantified by levels of peripheral MPO-DNA complexes) correlated positively to plasma syndecan-1 levels and plasma leakage, suggesting that degradation of EGL by neutrophils may contribute to vascular hyperpermeability [29]. In a mouse model, simultaneous inhibition of C-type lectin domain family 5 member A (CLEC5A, a pattern recognition receptor) and TLR 2, receptors involved in mediating neutrophil activation and NETs formation, significantly reduced vascular permeability and improved survival [30].

Despite much evidence linking activated neutrophils to vascular hyperpermeability, our current understanding on the roles of these immune cells in dengue, whether protective or pathological, is still limited. Previously, research on neutrophil functions in dengue has not been prioritised; this is potentially due to reports of neutropenia in DENV-infected patients [31]. However, emerging data strongly suggest that neutrophils are not merely bystanders during DENV infection, and future studies investigating the neutrophils-EGL axis in dengue-associated vascular leakage are needed.

## 4. Activated mast cells contribute to vascular hyperpermeability

Mast cells (MCs) are tissue-resident cells that are present at close proximity to the blood vessels. They have numerous cytoplasmic secretory granules that contain many inflammatory mediators including vasoactive molecules (Fig 1C). Like neutrophils, DENV stimulates MCs, and, upon activation, they will degranulate and release mediators such as chymase and tryptase; both enzymes have been shown to induce vascular permeability in vitro and in vivo [32,33]. Additionally, it was proposed that MC degranulation can be triggered by DENV-specific IgG interactions with FcγR, and this was associated with vascular hyperpermeability in mice [34]. Clinically, increased peripheral levels of chymase and tryptase were associated with vascular hyperpermeability compared to nonsevere dengue cases, and tryptase may be a more potent inducer of endothelial damage [32,33,35]. Vascular permeability was greatly reduced in MC-deficient mice compared with MC-replete controls, and drugs that stabilised MCs were observed to limit vascular leakage in DENV-infected mice [32,33]. Of clinical importance, nafamostat mesylate, a tryptase inhibitor, had a significant therapeutic effect in decreasing vascular hyperpermeability in DENV-infected mice, raising the possibility of it being an effective treatment for humans [32].

## 5. DENV-activated platelets contribute to vascular hyperpermeability

Platelets are small anucleated blood cells that are pivotal in maintaining vascular haemostasis. Interestingly, activated platelets and extracellular vesicles (EVs), released by DENV-activated platelets, can increase vascular permeability (Fig 1C) [36]. NS-1 was shown to activate platelets through TLR4, and knockout of TLR4 attenuated haemorrhaging in DENV-infected mice [37]. Furthermore, DENV can also activate platelets though C-type lectin-like receptors-2, which triggers the release of EVs. These EVs coactivate CLEC5A and TLR2 on neutrophils, inducing NETs formation that correlated with vascular hyperpermeability in mice [30]. DENV-induced EVs also enhance TNF-α secretion, another major mediator of vascular permeability [4]. In vitro, platelets incubated with both NS1 and neutrophils were more potent inducers of NETs formation, compared to stimulation with either NS1 or neutrophils alone [29]. Furthermore, cell-free histone H2A released from neutrophils has also been found to be increased in dengue patients, and H2A can increase platelet activation in vitro [38]. Whether activated platelets and their mediators directly contribute to vascular permeability is unknown, but evidence suggest a complex interaction with neutrophil and other immune cells to promote inflammation-associated vascular hyperpermeability [30,39].

## 6. Conclusions

Annually, approximately 390 million cases of DENV infections occur with approximately 96 million cases clinically symptomatic [40]. Dengue transmission may further increase due to a warmer climate globally in the coming decades. Currently, many of the mechanisms linking EGL, neutrophils, MC, and activated platelets to increased vascular permeability remain hypothetical, and more research are needed. EGL disruption affects vascular permeability, and therapeutic approaches to attenuate EGL degradation or enhance its synthesis should be explored. Neutrophils and MCs may serve a protective role during early DENV infection, but excess release of their mediators may cause immunopathology [26,33,41]. Activated platelets and EV may interact with various immunological cells to enhance inflammation, antibodies to disrupt these interactions may be useful in attenuating vascular pathologies observed. Delineation of the mechanisms that compromise glycocalyx integrity is required for rational development of targeted therapies to counteract the pathological responses of these immune cells in DENV infection.
